# Evaluation of the Mechanism of Jiedu Huazhuo Quyu Formula in Treating Wilson's Disease-Associated Liver Fibrosis by Network Pharmacology Analysis and Molecular Dynamics Simulation

**DOI:** 10.1155/2022/9363131

**Published:** 2022-06-06

**Authors:** Shao-Peng Huang, Sen Chen, Yan-Zhen Ma, An Zhou, Hui Jiang, Peng Wu

**Affiliations:** ^1^Experimental Center of Clinical Research, The First Affiliated Hospital of Anhui University of Chinese Medicine, Hefei, Anhui 230031, China; ^2^The Experimental Research Center, Anhui University of Chinese Medicine, Hefei, Anhui 230012, China; ^3^College of Integrated Traditional Chinese and Western Medicine, Anhui University of Chinese Medicine, Hefei, Anhui 230012, China

## Abstract

The Jiedu Huazhuo Quyu formula (JHQ) shows significant beneficial effects against liver fibrosis caused by Wilson's disease (WD). Hence, this study aimed to clarify the mechanisms of the JHQ treatment in WD-associated liver fibrosis. First, we collected 103 active compounds and 527 related targets of JHQ and 1187 targets related to WD-associated liver fibrosis from multiple databases. Next, 113 overlapping genes (OGEs) were obtained. Then, we built a protein-protein interaction (PPI) network with Cytoscape 3.7.2 software and performed the Gene Ontology (GO) term and Kyoto Encyclopedia of Gene and Genome (KEGG) pathway enrichment analyses with GENE DENOVO online sites. Furthermore, module analysis was performed, and the core target genes in the JHQ treatment of WD-associated liver fibrosis were obtained. Pathway and functional enrichment analyses, molecular docking studies, molecular dynamic (MD) simulation, and Western blot (WB) were then performed. The results indicated that 8 key active compounds including quercetin, luteolin, and obacunone in JHQ might affect the 6 core proteins including CXCL8, MAPK1, and AKT1 and 107 related signaling pathways including EGFR tyrosine kinase inhibitor resistance, Kaposi sarcoma-associated herpesvirus infection, and human cytomegalovirus infection signaling pathways to exhibit curative effects on WD-associated liver fibrosis. Mechanistically, JHQ might inhibit liver inflammatory processes and vascular hyperplasia, regulate the cell cycle, and suppress both the activation and proliferation of hepatic stellate cells (HSCs). This study provides novel insights for researchers to systematically explore the mechanism of JHQ in treating WD-associated liver fibrosis.

## 1. Introduction

Wilson's disease (WD), also termed hepatolenticular degeneration, is an autosomal recessive inherited fatal disorder with excessive copper accumulation [1]. Although the incidence and prevalence rates of WD in China are approximately 1.9/100,000 and 5.87/100,000, respectively, the absolute number of these patients should not be ignored, considering the large population base of China [2]. Studies have found that WD can further cause copper deposition in the liver and gradually progress from a mildly symptomatic liver inflammation to liver fibrosis even acute liver failure [3]. Accumulating research indicates that inflammation leads to activation of hepatic stellate cells (HSCs) and is a prominent driver of liver fibrosis [4, 5]. Some genes such as C-X-C motif chemokine ligand 8 (CXCL8), mitogen-activated protein kinase 1 (MAPK1), AKT serine/threonine kinase 1 (AKT1), SRC proto-oncogene (SRC), vascular endothelial growth factor A (VEGFA), and interleukin 6 (IL-6) have been reported to active EGFR tyrosine kinase inhibitor (EGFR-TKI) resistance, Kaposi sarcoma-associated herpesvirus (KSHV) infection, and human cytomegalovirus (HCMV) infection pathways, leading to liver inflammation and HSCs activation and promoting the progression of liver fibrosis [6–10]. In addition, existing therapeutic options are limited and mainly depend on copper chelation therapy [11]. At present, D-penicillamine (DPA) and trientine are the first-line therapeutic options for WD-associated liver fibrosis, but both have shortcomings [12]. DPA can cause worsening of neurological symptoms, myelotoxicity, and renal toxicity. Although trientine has few side effects, its curative effect is not significant [13, 14]. The Jiedu Huazhuo Quyu (JHQ) is a formula with intellectual property rights developed by the First Affiliated Hospital of the Anhui University of Chinese Medicine; its main herb constituents include Da Huang (Rhei Radix et Rhizoma), Huanglian (Coptidis Rhizoma), Danshen (Salviae miltiorrhizae Radix et Rhizoma), E Zhu (Curcumae Rhizoma), Jiang Huang (Curcumae Longae Rhizoma), and Jinqiancao (Lysimachiae Herba). JHQ has been clinically applied for almost 30 years which is not found to be toxic and side effects of human, and clinical application and animal experiments have been proven that it can improve liver function and WD-associated liver fibrosis [15, 16]. Although the clinical effect of JHQ is remarkable, the key components, core targets and main pharmacological mechanism of its prevention, and the treatment of WD-associated liver fibrosis have not been fully explained.

Therefore, in this study, we used network pharmacology, molecular docking, and molecular dynamics (MD) simulation to identify the key compounds and core targets of JHQ and further explored the potential mechanisms involved in the treatment of WD-associated liver fibrosis. Furthermore, we validated the results of network pharmacology in in vivo by western blot (WB) experiments.

## 2. Materials and Methods

**Figure 1:**
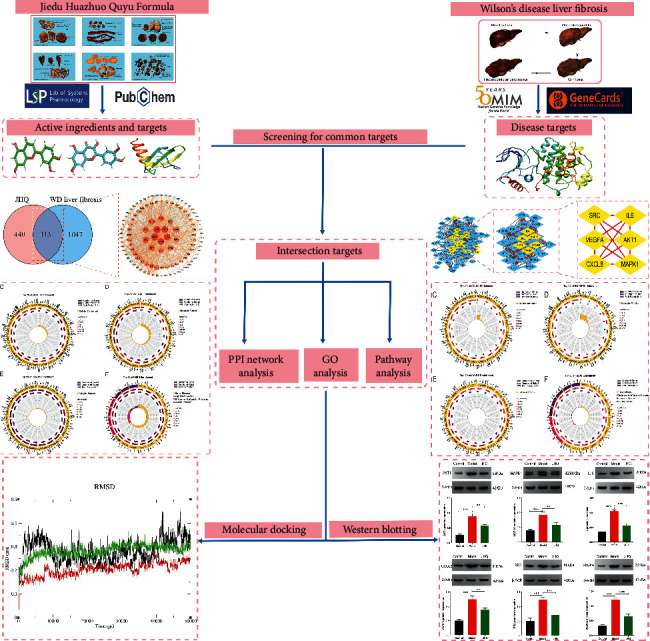
Schematic diagram of the network pharmacology approach to explore the mechanisms of JHQ in WD-associated liver fibrosis.

The strategy of this study is available in Figure 1.

### 2.1. Identification of Chemical Compounds in JHQ and Their Putative Targets

Compounds in the herbs in JHQ, including Da Huang, Huanglian, Danshen, E Zhu, Jiang Huang, and Jinqiancao, were searched in the TCMSP database (https://old.tcmsp-e.com/), and active compounds were mainly identified by integrated analysis of the pharmacokinetic properties of oral bioavailability (OB) ≥30% and drug likeness (DL) ≥0.18 [17–19]. An upset plot of compounds in the six herbs was generated by GENE DENOVO (https://www.omicshare.com) [20]. The prediction of putative targets of JHQ was conducted mainly with the TCMSP database. The SDF and mol2 format structures of some active compounds were uploaded into the Swiss Target Prediction (https://www.swisstargetprediction.ch/), DrugBank (https://go.drugbank.com/drugs), and PubChem database (https://pubchem.ncbi.nlm.nih.gov/) [21, 22]. The probability value of each potential target listed in the Swiss Target Prediction database was used to investigate the accuracy of current predictions, and other parameters were maintained as the defaults. The gene IDs of above targets were identified and standardized in the UniProt database (https://www.uniprot.org/) [23]. We used the retrieve/ID mapping tool in UniProt to convert the identifiers, which were of a different type, to UniProt identifiers.

### 2.2. Construction of a Protein-Protein Interaction (PPI) Network of Overlapping Genes (OGEs) and Enrichment Analysis

The target genes related to WD-associated liver fibrosis were retrieved from the GeneCards database (https://www.genecards.org/) and the OMIM database (https://omim.org/) [24, 25]. The search was conducted using the keywords “Liver fibrosis in hepatolenticular degeneration” and “Liver fibrosis in Wilson's disease.” The OGEs of putative targets of compounds and disease targets were visualized with the Venn tool of GENE DENOVO. We inputted all OGEs into the STRING database (https://stringdb.org/) with the confidence score set as ≥0.4, and we analyzed the PPI data and constructed the network with Cytoscape 3.7.2 [26, 27].

Then, Gene Ontology (GO) functional enrichment analysis included biological process (BP), molecular function (MF), and cellular component (CC) ontologies, and the Kyoto Encyclopedia of Genes and Genomes (KEGG) pathway enrichment which were visualized and analyzed by GENE DENOVO. The retrieval results were filtered with a threshold value of *P* < 0.05 and counted in descending order.

### 2.3. Screening of Core Target Genes and Enrichment Analysis

The molecular complex detection (MCODE) algorithm was used to develop the hub network to obtain clusters [28]. Genes in clusters established by MCODE are called hub targets. Then, we used the CytoNCA plugin to analyze the preliminary hub network to obtain network topological parameters such as betweenness centrality (BC), closeness centrality (CC), degree centrality (DC), local average connectivity (LAC), neighbor connectivity (NC), and subgraph centrality (SC) [29]. The computational equations and definitions of these parameters indicated the topological importance of nodes in the hub network. We took these nodes with top six BC, CC, DC, LAC, NC, and SC values to obtain the core network. We also performed GO enrichment and KEGG pathway analyses based on core genes obtained from module analysis.

### 2.4. Molecular Docking Simulation

Molecular docking simulation was performed to verify the critical components' binding ability to key targets and explore their accurate binding modes. The macromolecular protein target receptors were acquired from the RCSB PDB database (https://www.rcsb.org). Moreover, 2D structures of compounds were obtained from the TCMSP and PubChem databases. In addition, DPA is believed to be an effective first-line drug in the treatment of WD-associated liver fibrosis [13]. Docking analysis of DPA and its core targets was performed to obtain baseline affinity data, and the results showed that the values in the baseline groups were less than those for JHQ. The DPA data format was converted with Open Babel GUI software [30]. Then, molecular docking simulation was performed with the SwissDock online website (https://www.swissdock.ch/), and the results were visualized with PyMOL and Chimera1.14rc software [31–33]. In this analysis, the absolute value of the estimated value indicates the binding activity between a compound and protein; the greater the absolute value of the estimated value, the more stable is the binding of the compound to protein.

### 2.5. Simulation of Molecular Dynamics

The GROMACS package with the force field CHARMM was used for MD simulation. Simultaneously, the compound small-molecule ligands were uploaded to the CGenFF online tool (https://cgenff.paramchem.org/), and the CHARMM topology file was generated according to the CGenFF force field. Then, the MD simulation workflow includes four specific processes for minimizing energy, heating, balancing, and production dynamics. First, confine the heavy atoms (and small molecules) of proteins and perform 10,000 steps of energy minimization on the water molecule, then unlock the constraints, and perform 10,000 power minimization steps on the entire system. For energy optimization, the system was slowly heated to 300 K within 100 ps. After the heating was completed, the system was equilibrated for 100 ps under the npt ensemble. Finally, the system was subjected to 50 ns MD simulation under the npt ensemble. Trajectory data were saved every 10 ps. In order to calculate the binding affinity of three protein-ligand complexes, the g_mmpbsa tool was used to calculate the binding free energy of each complex. The specific formula is as follows:

(1) $$\begin{array}{c} \Delta \text{Gbind}=\Delta \text{EMM}+\Delta \text{Gsol}-T\Delta S,\\ \Delta \text{GMM}=\Delta \text{Einternal}+\Delta \text{Eelectrostatic}+\Delta \text{Evdw,}\\ \Delta \text{Gsol}=\Delta \text{GPB}+\Delta \text{GSA}. \end{array}$$

ΔEMM is composed of the electrostatic energy ΔEelectrostatic and van der Waals energy ΔEvdw, which is utilized to express the interaction energy between protein and its ligand. Meanwhile, ΔGsol contains two parts, where ΔGPB is the electrostatic solvation energy and ΔGSA is the nonpolar free solvation energy. Furthermore, TΔS is often ignored due to the low prediction accuracy, and the large amount of computation in the calculation process.

### 2.6. Core Protein Expression Levels Were Verified by WB

LX-2 cells were cultured in high-glucose DMEM medium (Solaibao, 12100) and maintained at 37°C in a humidified incubator in an atmosphere of 5% CO_2_. LX-2 cells were stimulated with 100 *μ*m CuSO_4_ for 48 h and then treated with 5% serum containing JHQ for 48 h. Total protein of the LX-2 cells sample was extracted by using RIPA lysis buffer (Biosharpc, BL504Ac) and centrifuged at 12000 rpm for 10 min. Total protein was separated by 10% SDS-polyacrylamide gel electrophoresis (SDS-PAGE) and was then transferred to nitrocellulose (NC) blotting membranes. After blocking with 5% nonfat milk for 2 h at room temperature, membranes were incubated with rabbit monoclonal anti-CXCL8 (1 : 500, Affinity Bioscience: #DF6998), anti-AKT1 (1 : 1000, Affinity Bioscience: #AF0836), anti-SRC (1 : 1000, Affinity Bioscience: #AF6161), anti-VEGFA (1 : 500, Affinity Bioscience: #AF5131), anti-IL-6 (1 : 500, Affinity Bioscience: #DF6087), rat monoclonal anti-MAPK1 (1 : 1000, Affinity Bioscience: #BF8004), and anti-*β*-actin (1 : 1000, Zs-BIO: #TA-09) primary antibodies overnight at 4°C and were then incubated with secondary antibodies at room temperature for 2 h, and detailed information is shown in Supplementary Table 1. The bands were detected by enhanced chemiluminescence (ECL) reagents, and the results are presented as the band density values. Densitometric quantification was performed using ImageJ software. Column bar graphs were obtained using GraphPad PRISM 8.

### 2.7. Statistical Analysis

Statistical analysis was performed using SPSS 23.0. At least three experiments were performed for each group, and all data are expressed as the mean ± standard deviation values. The mean value of three experiments performed for each group was used. The results of WB analysis were analyzed by one-way analysis of variance, and *P* < 0.05 was considered statistically significant.

## 3. Results

### 3.1. Compound-Putative Target Network

**Figure 2:**
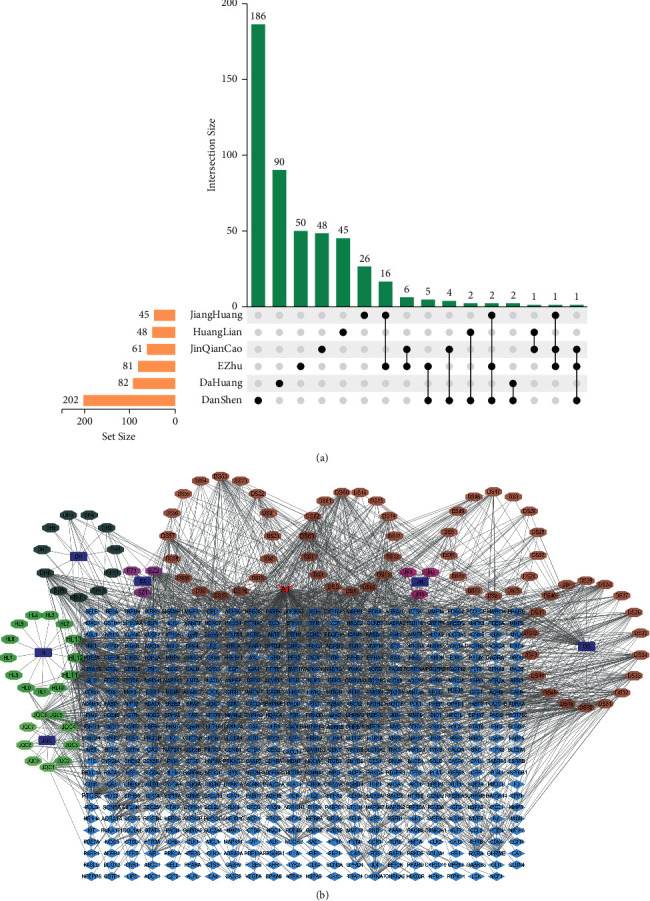
The number of overlapping ingredients and the herb-ingredient-target network relationships. (a) Venn diagram showing the number of overlapping ingredients among the six herbs. (b) Herb-ingredient-target network. Ellipses represent herbs in JHQ. Octagons represent active compounds in JHQ. Diamonds represent targets of active compounds. Edges represent interactions between compounds and targets vs. common targets of multiple herbs.

To construct the compound-putative target network of JHQ, we first analyzed 529 compounds of six herbs in JHQ (Figure 2(a)). Next, we screened the 103 potential active ingredients and 527 putative targets, and the interactions were identified and visualized as the herb-compound-target network, which contained 636 nodes and 2,060 edges in Cytoscape 3.7.2 (Figure 2(b)). Detailed information about this network is shown in Supplementary Table 2.

### 3.2. Identification of OGEs and Construction of the PPI Network

**Figure 3:**
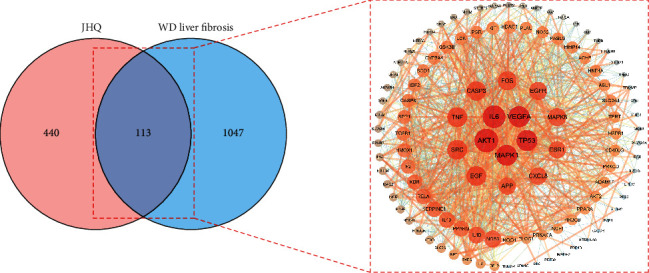
PPIs of 113 OGEs. (a) Venn diagram showing 113 common targets of JHQ and WD-associated liver fibrosis. (b) The PPI network: the larger the degree value is, the larger the node and the brighter the color. The larger the combined score is, the wider and brighter the line color is.

We analyzed and screened 499 targets and 742 targets of WD-associated liver fibrosis in the GeneCards database and the OMIM database, respectively. Subsequently, 1,187 disease targets were obtained after eliminating the overlap and taking the union of these two sets. Then, we intersected the JHQ and WD-associated liver fibrosis target genes and obtained 113 OGEs with the Venn diagram tool (Figure 3(a)); we imported OGEs into Cytoscape 3.7.2 to further obtain interaction networks (Figure 3(b)).

### 3.3. Enrichment Analysis

**Figure 4:**
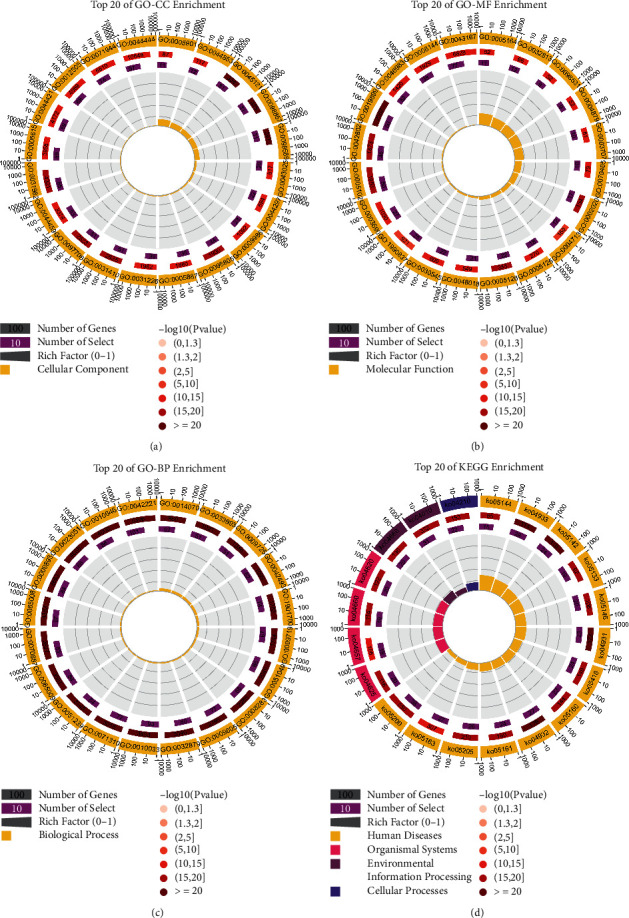
Enrichment analysis of 113 OGEs. (a) Top 20 enriched GO-CC terms. (b) Top 20 enriched GO-MF terms. (c) Top 20 enriched GO-BP terms. (d) Top 20 enriched KEGG pathways.

GO enrichment analysis revealed 6568 terms, among which 4611 were statistically significant. GO-CC enrichment analysis revealed 484 terms, among which 437 were statistically significant, and top 20 GO-CC terms are shown in Figure 4(a). GO-MF enrichment analysis revealed 686 terms, among which 484 terms were statistically significant, and top 20 GO-MF terms are shown in Figure 4(b). GO-BP enrichment analysis revealed 5398 terms, among which 3690 terms were statistically significant, and top 20 GO-BP terms are shown in Figure 4(c). KEGG enrichment analysis revealed 241 terms, among which 154 terms were statistically significant, and top 20 pathway entries are shown in Figure 4(d). Specific information about enrichment analysis is listed in Supplementary Table 3.

### 3.4. Screening of Core Target Genes and Functional Enrichment Analysis

**Figure 5:**
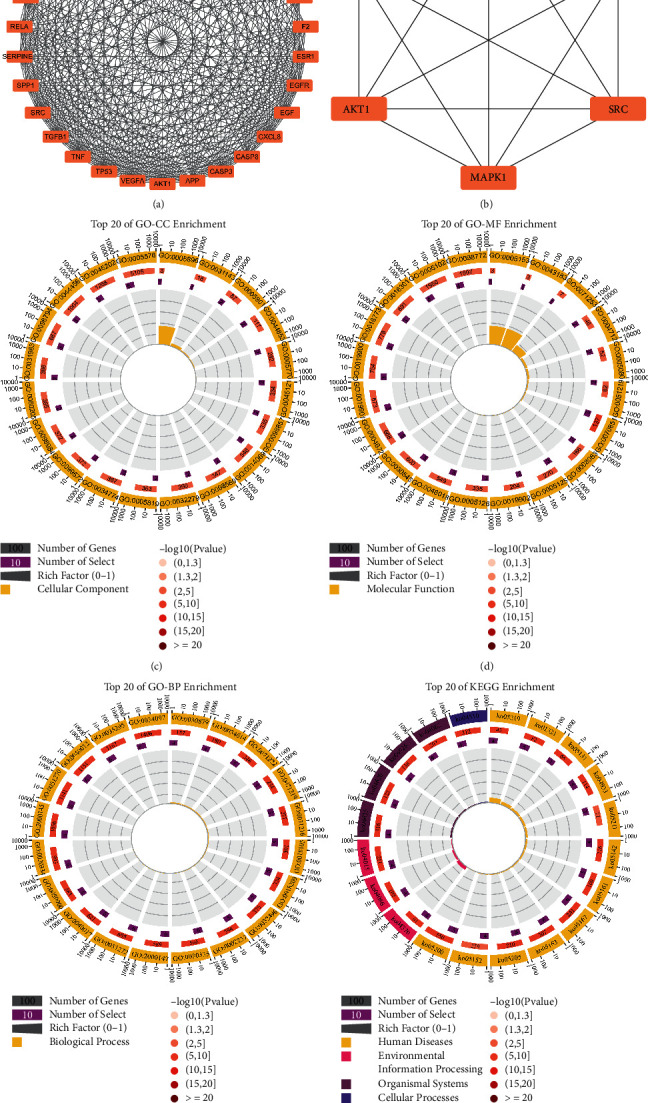
Identification of core target genes through module analysis adopted for gene set enrichment analysis of the 6 core target genes. (a) Module 2 consisted of 30 nodes and 378 edges as identified by the MCODE plugin in Cytoscape 3.7.2. (b) The top 6 core target genes identified by the CytoNCA plugin. (c) Top 20 enriched GO-CC terms. (d) Top 20 enriched GO-MF terms. (e) Top 20 enriched GO-BP terms. (f) Top 20 enriched KEGG pathways.

Module analysis with the MCODE plugin identified five modules, and we selected the hub module, which had an MCODE score of 26.07 [34]. The hub module of 30 key targets that we obtained was, especially, important in the treatment of WD-associated liver fibrosis by JHQ (Figure 5(a)). We selected the 6 most highly connected nodes (CXCL8, AKT1, MAPK1, SRC, VEGFA, and IL-6) as core networks with the CytoNCA plugin (Figure 5(b)). Functional enrichment analysis of 6 key target genes was performed after data screening to further understand the biological behaviors. Specific results of CytoNCA analysis are shown in Supplementary Table 4.

GO enrichment analysis revealed 1867 terms, among which 1742 terms were statistically significant. GO-CC enrichment analysis revealed 153 terms, among which 60 terms were statistically significant, and top 20 GO-CC terms are shown in Figure 5(c). GO-MF enrichment analysis revealed 113 terms, among which 81 terms were statistically significant, and top 20 GO-MF terms are shown in Figure 5(d). GO-BP enrichment analysis revealed 1964 terms, among which 1601 terms were statistically significant, and top 20 GO-BP terms are shown in Figure 5(e). KEGG enrichment analysis revealed 144 terms, among which 107 terms were statistically significant; top 20 pathway terms, such as EGFR tyrosine kinase inhibitor resistance, Kaposi sarcoma-associated herpesvirus infection, and human cytomegalovirus infection, are shown in Figure 5(f). Specific information about the enrichment analysis is listed in Supplementary Table 5.

### 3.5. Molecular Docking of Key Compounds and Core Target Genes

**Figure 6:**
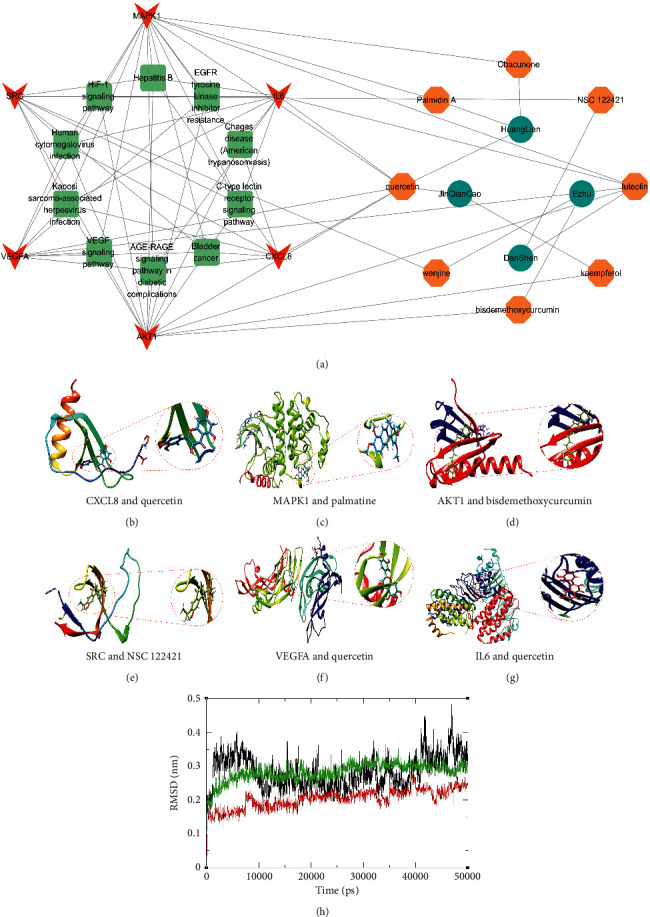
The herb-ingredient-target-pathway network relationship and the results of docking analysis and MD. (a) The herb-ingredient-target-pathway network. Octagons represent active compounds. Ellipses represent herbs. Rectangles represent pathways. Chevrons represent targets. (b) The molecular docking of CXCL8 to quercetin. (c) The molecular docking of MAPK1 to palmatine. (d) The molecular docking of AKT1 to bisdemethoxycurcumin. (e) The molecular docking of SRC to NSC122421. (f) The molecular docking of VEGFA to quercetin. (g) The molecular docking of IL-6 to quercetin. (h) RMSD plots for 3 protein-ligand complexes.

To better understand the interactions between JHQ and WD-associated liver fibrosis, we constructed an interaction network of the key herb-compound-gene-pathway interactions. It contains 8 key active components including quercetin, luteolin, obacunone, palmatine, kaempferol, bisdemethoxycurcumin, wenjine, and NSC 122421, 6 core genes including CXCL8, MAPK1, AKT1, SRC, VEGFA, and IL-6 (Figure 6(a)). We proposed that the eight components corresponding to the 6 core targets are the key components of JHQ. Docking analysis of proteins with compounds showed that proteins encoded by the core target genes interacted with the key compounds and the most likely active compounds, and that these compounds had stronger associations with core proteins than DPA (Figures 6(b)–6(g)). The results of molecular docking analysis are shown in Supplementary Table 6. Docking results of three best complexes (VEGFA-luteolin, Il-6-quercetin, and MAPK1-palmatine) were submitted to MD simulations.

### 3.6. MD Simulation

The MD simulation was performed using the VEGFA-luteolin, IL-6-quercetin, and MAPK1-palmatin complexes docked conformation to analyze the complex conformational dynamics. The stability of the system is checked by calculating the standard deviation of the simulated trajectory (RMSD) over a period of 50 ns. During the initial phase, the RMSD of protein-ligand complexes varies widely. At the end of simulation, the conformations of the three complexes reached equilibrium, and the structures were stable without obvious conformational fluctuations, as detailed in Figure 6(h), where VEGFA-luteolin (black lines) stabilized around 3.1 Å, VEGFA-luteolin (red lines) around 2.4 Å, and VEGFA-luteolin (green lines) around 2.9 Å. The binding free energies of the above three complexes were calculated by the MM-PBSA method (Table 1) and were −13.47 kcal/mol, −11.627 kcal/mol, and −10.892 kcal/mol. All three complexes showed good binding affinity, indicating their intrinsically high biological activity.

### 3.7. Core Target Protein Expression Was Examined by WB

**Figure 7:**
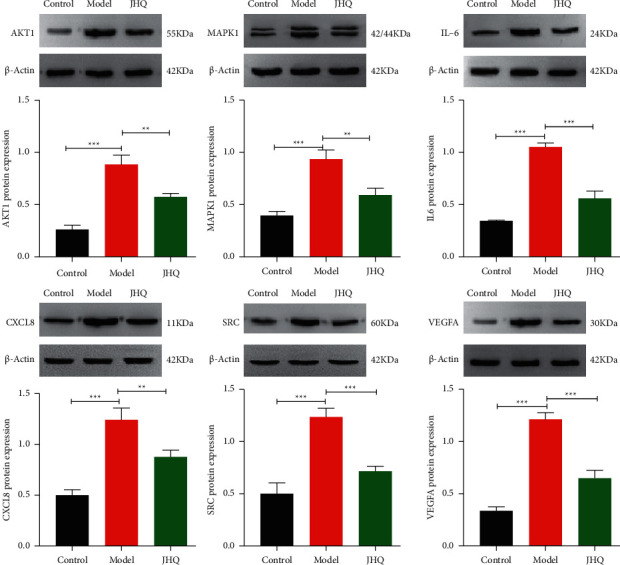
Effect of JHQ on core target proteins abundant in LX-2 cells after CuSO_4_ treatment. ^*∗∗∗*^*P* < 0.001 and ^*∗∗*^*P* < 0.01 compared with the model group.

As shown in Figure 7, to verify the results of network analysis, the expression levels of the core proteins AKT1, IL-6, CXCL8, VEGFA, SRC, and MAPK1 were determined by WB. The results showed that the expression levels of above core proteins were significantly higher in the model group than in the normal group, and that the levels of the core targets were significantly lower in the JHQ group than in the model group. This suggests that these core protein targets play essential roles in the effects of JHQ on reversing WD-associated liver fibrosis.

## 4. Discussion

As a new and comprehensive approach, network pharmacology, based on traditional pharmacology, bioinformatics, chemoinformatics, and network biology, can help researchers analyze the role of traditional Chinese medicine (TCM) in human biological networks from a global perspective. Because of the advantages of network pharmacology research strategies, new and innovative methods for the development of TCM have been opened.

In this study, using network pharmacological analysis, we found that JHQ contains a variety of active components that can inhibit WD-associated liver fibrosis development through multiple targets, biological processes, and related pathways. First, we searched databases for information on herbs and their associated targets and identified 103 active components (e.g., quercetin, luteolin, and obacunone) and 527 potential targets. These compounds were found to have enhanced liver regeneration, of epithelial-mesenchymal transition (EMT) inhibition, and antioxidative stress abilities. Quercetin, for example, ameliorates liver fibrosis by elevating the expression of the antioxidant transcription factor Nrf2 and alleviating the oxidative stress caused by excess copper in vivo [35, 36]. Luteolin exerts its therapeutic effect on liver fibrosis by promoting extracellular matrix degradation in fibrotic liver tissue and strongly enhancing the hepatic regeneration capability [37]. Obacunone is able to alleviate liver fibrosis by enhancing the antioxidant effects of glutathione peroxidase 4 (GPx-4) and inhibiting EMT [38]. These findings suggest that JHQ may exert its anti-liver fibrosis effect via these active compounds.

Immediately after this search, we obtained 1187 WD-associated liver fibrosis targets from the GeneCards and OMIM disease databases and identified 113 OGEs corresponding to active ingredients and disease targets. Then, module analysis of the 113 key OGEs was performed using MCODE and the CytoNCA plugin to obtain 6 core genes (CXCL8, MAPK1, AKT1, SRC, VEGFA, and IL-6), which might play important roles in the effects of JQH against WD-associated liver fibrosis. It has been reported that IL-6 and CXCL8 levels are related to the degree of liver fibrosis and can promote the process of liver fibrosis by inducing HSCs activation and regulating apoptosis [39–43]. It has also been shown that AKT regulates ECM degradation and that HSC activation aggravates liver fibrosis [44]. MAPK can induce liver inflammation and fibrosis, increase the extracellular matrix and affect the hypoxia inducible factor 1 subunit alpha (HIF-1*α*) signaling pathway to stimulate proliferation and activation of HSCs [45–49]. Other studies proved that SRC is involved in proliferation and activation of HSCs, impacts cellular autophagy, and induces the platelet derived growth factor receptor (PDGFR) and transforming growth factor beta (TGF-*β*) signaling cascades, thus accelerating the development of liver fibrosis [50–53]. VEGFA can induce proliferation and activation of HSCs and metastasis of cancer cells, stimulate neovascularization and extracellular matrix production, and facilitate progression of liver fibrosis and hepatocellular carcinoma development [54–57]. The above findings suggest that JHQ may regulate the expression of these key genes; inhibit HSCs proliferation and activation; suppress neovascularization, extracellular matrix production, and inflammatory reactions; and prevent liver fibrosis.

Next, by KEGG pathway enrichment analysis, we found that the 6 core target genes were mainly enriched in the EGFR tyrosine kinase inhibitor (EGFR-TKI) resistance, Kaposi sarcoma-associated herpesvirus (KSHV) infection, and human cytomegalovirus (HCMV) infection pathways. EGFR-TKIs can promote hepatocyte proliferation, cell cycle progression in hepatocytes, and angiogenesis through a variety of mechanisms involved in induction of HSCs proliferation and activation by core targets to promote the process of liver fibrosis [6, 7, 58, 59]. KSHV infection can cause KS, which is characterized by extensive neovascularization and inflammatory cell infiltration; induce vascular proliferation and inflammatory responses; inhibit apoptosis; and promote the development of liver fibrosis [8, 60, 61]. HCMV can affect the cell cycle, inhibit cell apoptosis, promote angiogenesis and HSC activation, and promote the process of liver fibrosis [9, 10, 62–64]. The above studies suggest that JHQ may exert beneficial effects by regulating the cell cycle and inhibiting inflammation, angiogenesis, and cancer progression in liver fibrosis patients.

Finally, to further validate the reliability of the target prediction results, molecular docking analysis and MD simulation were performed on the selected key compounds and core genes, and the results predicted that complexes had good binding affinity, indicating their intrinsically high biological activity. WB also showed that JHQ had a significant suppressive effect on the expression of the 6 core target proteins, suggesting that one of the possible mechanisms by which JHQ exerts its beneficial effects against WD-associated liver fibrosis is via the action of these active compounds on the 6 core target proteins.

## 5. Conclusion

In summary, the present study shows that the molecular mechanism of JHQ in the treatment of WD-association liver fibrosis was mainly achieved by active ingredients, such as quercetin, kaempferol, and triptolide. JHQ reduced the expression of CXCL8, MAPK1, AKT1, SRC, VEGFA, and IL-6 via EGFR tyrosine kinase inhibitor resistance, Kaposi sarcoma-associated herpesvirus infection, and human cytomegalovirus infection signaling pathways, thereby restricting the liver inflammation, neoangiogenesis, and activated HSCs in liver fibrosis. These findings could provide further insights into the mechanisms of CHM-based therapy in WD-associated liver fibrosis and assist in screening potential therapeutic targets in the future.

## Figures and Tables

**Table 1:** Contribution of various energy components of protein-ligand complex to binding free energy (kcal/mol).

Component	ΔEvdw	ΔEelectrostatic	ΔGPB/GB	ΔGSA	ΔEgas (EMM)	ΔGsol	ΔGbind
VEGFA-luteolin	−18.472	−2.494	9.265	−1.769	−22.021	6.904	−13.47
IL-6-quercetin	−23.264	−1.853	16.021	−2.531	−26.042	13.402	−11.627
MAPK1-palmatine	−19.065	−3.537	13.874	−2.164	−23.867	11.945	−10.892

ΔEvdw, van der waals energy; ΔEelectrostatic, electrostatic energy; ΔGPB/GB, polar solvation energy with the PB model; ΔGSA, nonpolar free solvation energy; ΔEgas (EMM), composed of the electrostatic energy ΔEelectrostatic, and van der waals energy ΔEvdw. ΔGsol, contains ΔGPB and ΔGSA; ΔGbind, the sum of ΔEvdw, ΔEelectrostatics, Δkeg/GB, and Δtakeoff.

## Data Availability

All the relevant data are provided within the paper and its supporting information files. The datasets analyzed during the current study are available from the corresponding author on reasonable request.

## Supplementary Materials

Supplementary Materials
*Supplementary Table 1.* Details for each antibody. *Supplementary Table 2.* Details for compound-putative target network. *Supplementary Table 3*. Enrichment analysis results of 113 genes. *Supplementary Table 4*. MCODE and CytoNCA analysis. *Supplementary Table 5*. Enrichment analysis results of 6 core genes. *Supplementary Table 6*. Molecular docking results.
